# The Effects of Physical Exercise on the Social Adaptation of Older Adults—With Reference to the Mediating Effect of Aging Identity

**DOI:** 10.3390/bs15111491

**Published:** 2025-10-31

**Authors:** Zhiming Zhang, Jiaxiang Zhang, Cheng Fu, Chengwen Fan

**Affiliations:** 1 College of Physical Education, Hunan University of Technology, Zhuzhou 412007, China; m22045201027@stu.hut.edu.cn (Z.Z.); 14581@hut.edu.cn (C.F.); 2 School of Journalism and Communication, Shanghai University of Sport, Shanghai 200438, China; 2511415007@sus.edu.cn

**Keywords:** physical exercise, social adaptation, aging identity, older adults, CLASS

## Abstract

Maintaining social adaptation in later life has become a key challenge amid China’s rapidly aging population. Using nationally representative data from the China Longitudinal Aging Social Survey (CLASS 2023), this study examined the relationship between physical exercise and social adaptation among 8913 older adults. Ordinary least squares regression and the Karlson–Holm–Breen decomposition method were applied to test both direct and mediating effects. The results showed that physical exercise significantly improved social adaptation (β = 0.452, *p* < 0.001), while aging identity played a partial mediating role, accounting for approximately 11.0% of the total effect. The association was stronger among those aged 80 and above, with lower education and income, without chronic diseases, and covered by social security. These findings suggest that physical exercise enhances social adaptation not only through physical benefits but also by strengthening psychological resilience and fostering a positive sense of aging, providing valuable evidence for developing inclusive aging policies and targeted exercise interventions.

## 1. Introduction

According to data from China’s Seventh National Population Census, the total number of people aged 60 years and above has exceeded 260 million, accounting for 18.70% of the total population—an increase of 5.44 percentage points compared with the Sixth Census. Among them, 190 million are aged 65 and above, accounting for 13.5% of the population (National Bureau of Statistics of China, 2021). With the accelerating process of population aging, the issue of social adaptation among older adults has become an increasingly prominent social concern (Grech, 2019). Upon entering later life, individuals face multiple transitions, and their capacity for social adaptation directly affects their quality of life and degree of social integration. Poor social adaptation can lead to anxiety, depression, and other emotional problems (He et al., 2021) and may even undermine family support systems and the stability of social structures (Tsai, 2017).

As one of the driving forces of social evolution (Spencer, 1898), social adaptation refers to the process by which individuals adjust to changes in their social environment through modifications in behavior, cognition, and emotion (Rachmad, 2022). As a key indicator of whether older adults can cope with environmental change and integrate into social life (Gai & Mei, 2024), social adaptation has gradually become a central topic in active aging research. As early as 1946, the World Health Organization (WHO) emphasized that health encompasses not only physical and psychological well-being but also the ability to adapt socially (Grad, 2002). Against this backdrop, identifying scientific and effective interventions to enhance social adaptation among older adults has become an urgent research agenda.

Physical exercise, as a sustainable and health-promoting lifestyle, has been widely recognized for its positive effects on improving physical function, alleviating negative emotions, and promoting social participation (X. Li & Li, 2025; McAuley et al., 2005; Netz et al., 2005; X. Ma et al., 2025; C. Li et al., 2023). Existing research indicates that regular physical exercise not only enhances physiological functioning among older adults but also improves social functioning and subjective well-being by strengthening self-efficacy and emotional regulation (Caspersen et al., 1985; de Souto Barreto, 2009; Paterson & Warburton, 2010). However, current studies still exhibit two main limitations: first, the psychosocial mechanisms through which physical exercise enhances social adaptation during later life—the “period of self-identity reconstruction” (Robbert, 1983)—have not been adequately explored; second, most empirical studies have relied on regional or small-scale samples, or mixed-age groups, lacking systematic and mechanism-based analysis for older adults. Particularly in the context of China’s rapidly aging society, whether and how physical exercise can improve social adaptation through psychosocial pathways remains to be verified using robust empirical evidence.

In recent years, the concept of aging identity—defined as an individual’s subjective perception and evaluation of their own aging process—has emerged as a key psychological construct in gerontological research. Specifically, aging identity concerns how old or young a person feels compared to their chronological age (Barrett & Barbee, 2022). In this study, higher values of aging identity indicate a stronger sense of resistance to aging (that is, perceiving oneself as younger or “not yet old”), whereas lower values indicate greater acceptance of aging. Prior studies have shown that physical exercise can strengthen older adults’ sense of vitality and control, thereby delaying their subjective perception of aging and shaping a more positive aging identity (Diehl et al., 2014; Guo & Huang, 2025; Kim et al., 2016). Conversely, a more positive aging identity enhances social interaction and adaptability (Levy & Myers, 2004; Jiang & Shi, 2024). Based on these findings, it can be inferred that the promotion of social adaptation through physical exercise is not entirely direct but may be partially mediated by aging identity (W. Liu & Qi, 2024; Putnam, 1995).

Therefore, drawing on data from the 2023 China Longitudinal Aging Social Survey (CLASS 2023), this study systematically examines the relationship between physical exercise and social adaptation among older adults and introduces aging identity as a mediating variable to explore the underlying psychological mechanism. In addition, the study analyzes the heterogeneity of this relationship across different age groups, education levels, income brackets, health conditions, and social security statuses. The findings aim to provide both theoretical insight and practical guidance for promoting healthy aging and enhancing social functionality among older adults. Based on previous literature and theoretical reasoning, the following hypotheses are proposed:

**H1:**  *Physical exercise significantly enhances the level of social adaptation among older adults.*

**H2:**  *Physical exercise significantly enhances positive age identity.*

**H3:**  *A more positive age identity significantly improves social adaptation.*

**H4:**  *Aging identity mediates the relationship between physical exercise and social adaptation.*

## 2. Methods

### 2.1. Data Sources

The data used in this study come from the 2023 China Longitudinal Aging Social Survey (CLASS2023), a large-scale nationwide and continuous social survey project jointly designed and implemented by the Center for Population and Development Studies and the Institute of Gerontology of Renmin University of China. CLASS2023 covers 464 village (neighborhood) committees in 28 provinces (autonomous regions and municipalities directly under the central government) and collects samples from nearly 12,000 older adults, covering multidimensional information on health, lifestyle, psychological status, social relationships, subjective feelings, etc., which is highly representative.

As this study focused on the relationship between physical exercise and the social adaptation of older adults, which involves corresponding cognitive topics, to achieve data quality control, the study referred to the treatment of the MMSE scale, and samples that scored less than 3 out of 5 cognitive questions (Folstein et al., 1975) were excluded (*n* = 323). Individuals who did not complete the social adaptation, aging identity, or physical exercise questionnaires were excluded (*n* = 1251). The sample of data with one or more control variables missing was approximately 1180 cases, and the final sample consisted of 8913 participants (mean age 71.29 years, SD = 6.00; 52.19% male, 47.81% female).

### 2.2. Variable Measurement

#### 2.2.1. Dependent Variable: Social Adaptation

The dependent variable, social adaptation, was measured using the Social Adaptation Scale developed by B. Chen (2008), as included in the E6 module of the CLASS 2023. CLASS is a nationally representative longitudinal database widely used in gerontological and sociological research in China, ensuring the authority and reliability of the measurement data. The scale comprehensively evaluates older adults’ subjective perceptions of social participation, self-worth, learning interest, and adaptability to social change, reflecting their ability to maintain balance between individual and social environments.

Specifically, the eight items are as follows: (1) “If given the opportunity, I am willing to participate in some village or community committee work”; (2) “I often think about what I can do for society again”; (3) “I enjoy learning at present”; (4) “I feel that I am still a useful person to society”; (5) “Social changes are happening so fast that I find it difficult to adapt to them”; (6) “More and more opinions are now difficult for me to accept”; (7) “The newer the social policies become, the harder they are for me to accept”; and (8) “Current social changes are increasingly unfavorable to older people.”

Each item was rated on a five-point Likert scale (1 = strongly disagree, 5 = strongly agree). Among them, Items 5–8 were reverse coded to ensure that higher total scores consistently reflected better social adaptation. The total score ranges from 8 to 40, with higher scores indicating a higher level of social adaptation. In this study, the internal consistency of the scale was satisfactory (Cronbach’s α = 0.747), demonstrating good reliability and internal structural stability. The use of the standardized CLASS questionnaire ensures high-quality, comparable data across studies and provides a valid basis for the measurement of social adaptation among older adults in China.

#### 2.2.2. Independent Variable: Physical Exercise

This study focuses on whether older adults participate in physical activity on a regular basis. The responses to the questionnaire “How often do you participate in physical activity?” were binarized in consideration of data availability and in accordance with the practice of relevant academic research. The participants who answered “less than once a month on average”, a very low-frequency option, were assigned a value of 0, whereas the participants who answered all other options were assigned a uniform value of 1, which means that they participated in physical activity.

#### 2.2.3. Mediating Variable: Age Identity

The mediating variable in this study was aging identity. Here, aging identity refers to the difference between how old respondents feel (‘perceived age,’ from the question “How old do you consider yourself to be?”) and their actual chronological age (“What year were you born?”). This difference reflects an individual’s subjective perception of their own aging status in relation to their actual age. A positive score indicates that individuals perceive themselves as younger than their actual age, representing a stronger and more positive aging identity. In contrast, a lower or negative score reflects a greater acceptance of aging or a feeling of being older than one’s chronological age. This measure captures the psychological dimension of aging, emphasizing how individuals interpret and internalize the aging process rather than their objective age.

#### 2.2.4. Control Variables

With reference to existing studies, the control variables in this study incorporated several key individual and family social characteristics that affect social adaptation, such as gender, age, type of residence, years of education, presence of a spouse, being an empty nester, presence of chronic diseases, number of children, and presence of social security. To measure the quantitative characteristics of educational accumulation more scientifically in statistical modeling, educational attainment was converted into a yearly variable. In the question “What is your educational level?” The seven answers of “illiterate”, “private school/literacy class”, “primary school”, “junior high school”, “senior high school/secondary school”, “junior college”, “college” and “bachelor’s degree and above” were assigned values of 0, 3, 6, 9, 12, 15, and 16, respectively. (See Table 1 for variable selection).

### 2.3. Methods of Analysis

In this study, Stata 17.0 software was chosen to statistically analyze the sample data. First, multiple models were used to fit the data according to the research needs and dependent variable attributes. First, the ordinary least squares (OLS) method was used to construct a linear regression model for fitting analysis to verify the significance and explanatory power of H1. Model 1 is as follows:

(1) $${SocialAdapt}_{i}=\beta_{0}+\beta_{1}\cdot {Exercise}_{i}+\beta_{2}X_{i}+\epsilon_{i}$$

where ${SocialAdapt}_{i}$ is the social adaptation score of individual i; ${Exercise}_{i}$ represents the dummy variable of whether or not to participate in physical exercise; $X_{i}$ denotes a series of individual and family social characteristics such as gender, age, years of education, marital status, chronic diseases, number of children, social security, etc.; $\epsilon_{i}$ denotes the error term obeying a normal distribution with zero mean and homoscedasticity; and $\beta_{0}$, $\beta_{1}$, and $\beta_{2}$ represent the parameters to be estimated.

Second, we aimed to verify whether “aging identity” is a mediating mechanism between “physical exercise” and “social adaptation” in older adults (H4). In this study, we used the stepwise regression method proposed by Baron and Kenny (1986) to carry out the mediation test. The mediation effect model is constructed on the basis of Equation (1), and the effect of “physical exercise” on “aging identity” is fitted by the least squares model (OLS model) to verify the significance of H2. Then, the variable “aging identity” is introduced into Model 1 to test whether its effect on social adaptation is significant (H3). Model 2 and Model 3 are constructed as follows:

(2) $${AgingIdentity}_{i}=\beta_{0}+\beta_{1}\cdot {Exercise}_{i}+\beta_{2}X_{i}+\epsilon_{i}$$

(3) $${SocialAdapt}_{i}=\beta_{0}+\beta_{1}\cdot {Exercise}_{i}+\beta_{2}\cdot {AgingIdentity}_{i}+\beta_{3}\cdot X_{i}+\epsilon_{i}$$

where ${AgingIdentity}_{i}$ is the mediating variable, and the other variables and parameters are the same as above. After verifying that the causal path structure is significantly established, a mediation analysis method is formally introduced to identify whether physical exercise indirectly affects the social adaptation of older adults by influencing their self-perception of age. In this study, the KHB method (Karlson–Holm–Breen method) was used to decompose the mediating effect, which is suitable for identifying the mediating mechanism in both nonlinear and linear models and can accurately differentiate between the direct effect, the indirect effect, and the total effect and avoid estimation bias caused by the introduction of control variables.

To test the robustness and reliability of the results, the results of the study were further evaluated via the sample replacement method test, model substitution method test and bootstrap method test to eliminate estimation bias due to possible endogenous disturbances.

## 3. Results

### 3.1. Descriptive Statistical Analysis

The results of the descriptive statistics for each variable are shown in Table 2. Among the 8913 older adults surveyed, there was a certain difference in social adaptation between those who participated in physical exercise (mean 24.63) and those who did not (mean 23.98), indicating that physical exercise may have a positive effect on the social adaptation of older adults. In addition, the gender distribution of the older adults in the sample was balanced: the average age was 71.29 years, the overall education level was low, and most of the older adults enjoyed old-age security. The specific statistical results are shown in Table 2.

**Figure 1:**
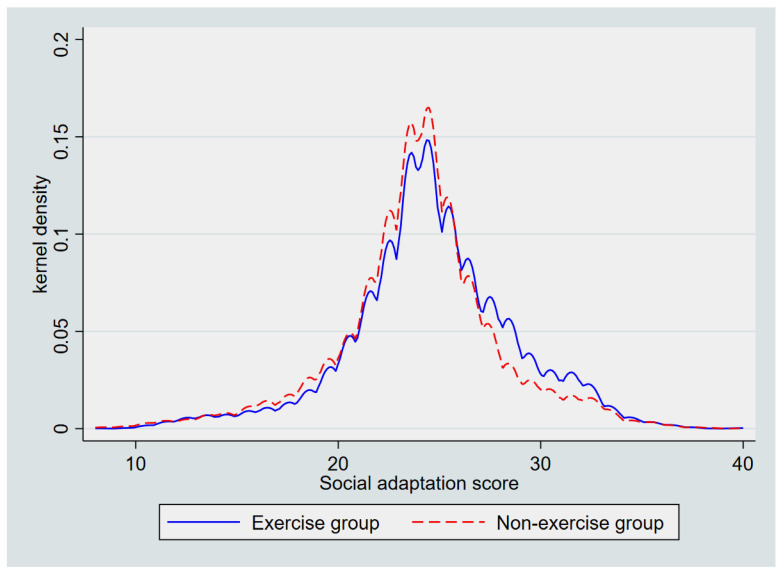
Kernel density distribution of social adaptation among older adults by physical exercise participation (N = 8913).

To further examine the distributional differences in social adaptation between older adults who participated in physical exercise and those who did not, kernel density estimation (KDE) was applied, and the results are presented in Figure 1. The *x*-axis represents the total social adaptation score (range = 8–40), and the *y*-axis shows the estimated probability density. As shown in the figure, the distribution of the exercise group (solid blue line) is slightly right-skewed, with a longer upper tail and a higher density in the middle- to high-score range (approximately 25–35), indicating generally higher levels of social adaptation. In contrast, the non-exercise group (dashed red line) exhibits a more peaked and centralized distribution, suggesting that their scores were more concentrated in the moderate to lower range. These visually distinct patterns indicate that participation in physical exercise is associated with higher social adaptation among older adults.

To further verify the relationships among the main variables, a correlation analysis was conducted on physical exercise, aging identity, and social adaptation (Table 3). The results revealed that physical exercise was significantly and positively correlated with aging identity (r = 0.118, *p* < 0.01) and social adaptation (r = 0.081, *p* < 0.01). In addition, aging identity was significantly associated with social adaptation (r = 0.169, *p* < 0.01). These findings preliminarily indicate that physical exercise and aging identity are positively linked to social adaptation among older adults, supporting the hypothesized mediating framework and providing a foundation for subsequent regression and mediation analyses.

### 3.2. Main Effects Analysis: The Impact of Physical Exercise on Social Adaptation

In this study, the relationship between the physical exercise and social adaptation of older adults was analyzed through the OLS method, as shown in Table 4. The results of the model’s analysis revealed that physical exercise has a significant positive effect on the social adaptation of the elderly (β = 0.452, *p* < 0.001), i.e., after controlling for age, gender, income, education, marital status, and health status, the elderly who participate in physical exercise show a greater level of social adaptation. In addition, income level (β = 0.343, *p* < 0.001) and years of education (β = 0.113, *p* < 0.001) likewise had a significant positive effect on social adaptation, whereas age (β = −0.029, *p* = 0.001) and empty nest status (β = −0.396, *p* = 0.041) significantly negatively affected social adaptation. The variables of sex, marital status, chronic diseases, social security and number of children did not have a significant effect. The coefficient of determination of the model, R^2^, was 0.0447, which is consistent with the common level of explanatory power of behavioral variables in social science research. Hypothesis H1 was tested in the experiment.

### 3.3. Analysis of Mediating Effects: The Role of Aging Identity in the Relationship Between Exercise and Social Adaptation

To further explore the role of the effect of physical exercise on the social adaptability of older adults, this study explored whether age-related identity plays a mediating role between physical exercise and social adaptation through mediation effect analysis. To ensure the rigor of the analysis, we first verified the effect of physical exercise on the age identity of older adults through a least squares regression model, and the results are shown in Table 5. Physical exercise has a significant positive effect on the age identity of older adults, and the coefficient of the core explanatory variable is 1.315; i.e., the age identity of older adults who participate in physical exercise significantly increases by 1.315 units, and older adults who participate in physical exercise are more inclined to “defy old age” and have a more positive age identity. This result verifies H2 and provides a theoretical and data basis for establishing the mediating effect path.

To test the effect of aging identity on the social adaptability of older adults (H3), this study continued to construct OLS Model 3 on the basis of Model 1 and introduced the aging identity variable while controlling for physical exercise and demographic variables. As shown in Table 6, the model revealed that aging identity had a significant positive effect on social adaptation (β = 0.038, *p* < 0.001), supporting its validity as a mediating variable. Specifically, the more positive the aging identity—meaning the older adults perceived themselves as younger or held a more affirmative attitude toward aging—the higher their level of social adaptation, and hypothesis H3 was verified. Moreover, the coefficient of the core explanatory variable of the effect of physical exercise decreased from 0.452 to 0.403 after the addition of age, but the result was still significant (*p* < 0.001). At the same time, the R^2^ of the model’s explanatory power increased to 0.2565, which indicated that physical exercise still had an independent influence after controlling for the mediator variable. From the results of Model 1 and Model 3, the stepwise analysis of the social adaptability of the elderly initially indicated that the age-related identity variable had mediating potential, supporting further analysis of the decomposition of the mediating effect.

To more scientifically and rigorously verify the mediating role of age identity in the relationship between physical exercise and social adaptation in older adults, this study further used the KHB method to decompose and analyze the model, and the analysis results are shown in Table 7. In the reduced model without the mediating variable, the coefficient of physical exercise’s influence on older adults’ social adaptation was 0.452 (*p* < 0.001), whereas in the full model after the introduction of age, the coefficient decreased to 0.403 (*p* < 0.001), and the difference between the coefficients of the two models was 0.050 (*p* < 0.001), with a confidence interval of [0.029, 0.069], suggesting that part of the effect of exercise may be realized indirectly through the mediating variable. H4 was verified through the above rigorous experimental steps, confirming that age identity plays a partial mediating role in the effect of physical exercise on social adaptation.

### 3.4. Robustness Test

There are three parts of the robustness test in this study: the sample replacement method test, bootstrap method test and model substitution method test. In the sample replacement test, the 2020 China Longitudinal Aging Social Survey (CLASS2020) is chosen as the source of data for the sample replacement test; 8478 samples are obtained after the data are cleaned, and the OLS estimation for the control group is continued through the same modeling method. The results are shown in Table 8. Physical exercise in the model still has a highly significant positive effect on the social adaptation of older adults, in which the coefficient of the core explanatory variable is 0.554, indicating that this core explanatory variable is robust across different samples. Moreover, the explanatory power of aging identity as a mediator variable is also consistently significant in both models, indicating that the mediating path logic that physical exercise enhances social adaptation by improving aging identity also holds after the sample change.

Next, the KHB mediation effect analysis was repeated for the control group. As shown in Table 9, the coefficient of the effect of physical exercise on social adaptation was 0.652 (*p* < 0.001) without the introduction of the mediator variable “aging identity”. It decreased to 0.554 (*p* < 0.001) after the introduction of the mediating variable, with a difference of 0.099 (*p* < 0.001) and a confidence interval of [0.066, 0.132]. This result reaffirms the significant partial mediating role of age in the influence of physical exercise on social adaptation and strengthens the robustness of this study.

In the model substitution test, since the social adaptation scale consists of five cumulative Likert-type entries, which essentially originates from the accumulation of ordered categorical variables, the ordered logit regression model that preserves the order of response options (Liddell & Kruschke, 2018) for hierarchical data was chosen to replace the OLS model for estimation, and the results are shown in Table 10. Physical exercise (OR = 1.224, *p* < 0.001) continued to have a significant positive effect on the level of social adaptation of older adults after controlling for other variables. Age also had a significant effect on identity (OR = 1.013, *p* < 0.001), indicating that its positive effect on social adaptation remains. Although the mode of interpretation of the variables changed from linear to probability ratios, the core conclusions remained unchanged, verifying the robustness of the modeling setup, and H1, H3, and H4 were once again supported.

To ensure the robustness of the mediation results, this study further applied a bootstrapping test with 5000 resamplings, and the results are presented in Table 11. The analysis revealed that physical exercise was positively associated with social adaptation through aging identity, with an indirect effect of 0.0499 and a bootstrapped 95% confidence interval of [0.0292, 0.0697], which does not contain zero. This confirms that the indirect association consistent with mediation is statistically significant. Further calculations indicated that this indirect pathway accounted for approximately 11.04% of the total association, reinforcing the conclusion that aging identity statistically mediates part of the link between physical exercise and social adaptation. The results are highly consistent with those obtained from the KHB method, jointly confirming the reliability and robustness of the findings. Nevertheless, given the cross-sectional design, these findings should be interpreted with caution—they reflect a statistical association rather than a verified causal mediation process. Future research employing longitudinal or experimental data would be valuable for confirming the temporal order and validating the psychological mechanism identified in this study.

### 3.5. Heterogeneity Analysis

To further identify the differential effects of physical exercise on the social adaptation of different groups of older adults, this study, on the basis of clarifying the domain-wide effects, conducts group regressions according to age group, education level, urban/rural distribution, income level, health status, and old-age security to further elucidate the group differences in the social adaptation of older adults due to physical exercise.

Table 12 reveals the differences between groups of older persons of different ages and levels of education. In terms of the age variable, physical exercise has a significant enhancing effect on the social adaptability of all age groups, in which the value of the coefficient of the core explanatory variable tends to increase as age increases, especially in the 80-year-old and above group, whose value reaches 0.757, which is the highest among all age subgroups and is higher than that of the 60–69-year-old group by approximately 82%. The reason for this finding may be that senior citizens face more physical deterioration and social role changes, and physical exercise can be a positive way of coping, significantly helping them alleviate physical and mental stress and enhancing their sense of social participation and self-worth identity. The results of grouping by education level also revealed significant differences in the effects of physical exercise on the social adaptability of older adults with different education levels. For the illiterate older adult group, physical exercise had the greatest impact, with a regression coefficient of 0.892, indicating that this group has a particularly positive attitude toward physical exercise. The likely reason for this is that lower-educated older adults tend to have more limited access to information, and physical exercise becomes an important way for them to improve their physical and mental health and enhance their sense of social participation. However, for the group with higher education, the regression coefficients or significance fell to some extent, showing some diminishing marginal utility effect. Among them, the regression coefficient for the group with primary and secondary education is 0.531, whereas the significance level for the group of older adults with tertiary education and above has decreased (*p* < 0.05), probably because the increase in education is accompanied by higher health awareness and the formation of regular exercise habits in this group, so the impact of physical exercise on them is smoother and there is less room for the enhancement of utility.

Owing to differences in the economic base and regional resources, the social adaptability caused by physical exercise also differs across income levels and urban and rural residences of the older adult population. Table 13 shows that physical exercise significantly (*p* < 0.01) enhances the social adaptation of older adult groups with different income levels, but the regression coefficient for the low-income group reaches 0.663, which is much greater than that of the high-income group (0.241), suggesting that the enhancement of the social adaptation of the low-income elderly group through physical exercise is more obvious. The low-income group usually faces more life pressure, and low-income groups usually face more life pressure and resource constraints. Physical exercise becomes an important way for them to improve their physical and mental health and enhance their social connections. Although the high-income group also benefits from physical exercise, the marginal contribution of physical exercise to their social adaptability is relatively small because they have more social support and health management tools. In terms of urban‒rural differences, the regression coefficient of the urban household group (0.536) is significantly greater than that of the nonurban household group (0.399), indicating that the social adaptation of the urban elderly group benefits more from physical exercise, a phenomenon that may be closely related to the differences in urban and rural resources and infrastructures, with the effect of physical exercise being more pronounced in the urban elderly group because of a more complete health management and social support system. While the rural elderly group benefits from the enhancement effect of physical exercise, the benefits of physical exercise are relatively weak due to the lack of resources.

Table 14 shows that physical exercise significantly contributes to the social adaptation of different groups of older adults with chronic disease status, with a β value of 0.554 for older adults without chronic disease being higher than the value of 0.429 for the group of older adults with chronic disease, suggesting that the enhancement of social adaptation caused by physical exercise is more pronounced in the case of healthy older adults. This may be related to the fact that healthy older adults have more advantages in terms of physical function and mobility, and healthy older adults are able to participate more fully in various forms of physical exercise, thus obtaining greater psychological and social benefits. For the group of older people suffering from chronic diseases, there may be limitations in the intensity and frequency of exercise due to the disease, which in turn affects the effectiveness of physical exercise promotion. For the old-age security component, the elderly group with old-age security showed a significant positive effect on the promotion of social adaptability through physical exercise, with a regression coefficient of 0.562, indicating that physical exercise can increase social adaptability by 0.562 units. This is because old-age security provides them with financial support and social security, which enables this group to participate in physical activities with greater peace of mind, thus effectively improving their social adaptability. In contrast, the regression coefficient for the elderly group without old-age security is −0.829 (*p* < 0.05), indicating that physical exercise in the elderly group without old-age security has a negative effect on social adaptability. This negative effect may be related to the fact that older people without old-age security face greater economic pressure, health instability and life uncertainty, which lead to their lower participation in physical exercise, thus affecting the effect of exercise on social adaptability.

## 4. Discussion

Using the CLASS2023 sample pool as a data source, this study systematically examined the direct and indirect mechanisms of the impact of physical exercise on older adults’ social adaptation levels. It verified that physical exercise not only significantly enhances older adults’ social adaptation levels but also predicts older adults’ social adaptation by partially mediating the enhancement of their age-of-age identity. The relationship between physical exercise and social adaptation of older adults was further explained.

### 4.1. Physical Exercise and Social Adaptation

This study revealed that physical exercise has a significant positive effect on the social adaptation of older adults, resulting in a 0.4523 unit increase in the level of adjustment. Research hypothesis H1 was verified. This finding aligns closely with previous studies. For example, X. Liu et al. (2022) reported that individuals who lack engagement in physical exercise tend to develop negative self-perceptions and avoidance behaviors, which in turn increase social isolation. In contrast, regular participation in exercise promotes positive social experiences and strengthens interpersonal confidence and competence (X. Liu et al., 2022). W. Ma (2017) also found that the level of physical participation is highly correlated with individuals’ social adaptation, and that appropriate exercise can enhance emotional regulation and social interaction. From the perspective of self-efficacy theory, physical exercise enhances individuals’ sense of control and perceived competence, enabling them to cope with social changes more effectively and thereby improve their level of social adaptation (Gordon et al., 2020). S.-P. Chen et al. (2006) further demonstrated that a higher frequency of exercise participation corresponds to better performance in the domain of social adaptation. Together, these results reveal the psychological mechanisms through which physical exercise contributes to improved social adaptation.

At the same time, personality psychology suggests that physical exercise, as a process of strengthening self-discipline and emotional regulation, enables individuals to respond to environmental changes and social challenges more steadily, thereby indirectly enhancing their social resilience and overall quality of life (Zhou et al., 2024). Physical exercise is not only a behavioral practice that promotes physical health but also a comprehensive activity embedded within a social context. In practice, participation in exercise is often accompanied by multiple functions such as emotional regulation, interpersonal interaction, and information exchange. As Hu and Wang (2017) found, physical exercise enhances social adaptability by improving self-esteem, facilitating the acquisition of social support, and fostering better interpersonal relationships. Similarly, VanKim and Nelson (2013) demonstrated that individuals who regularly engage in physical exercise exhibit stronger interpersonal management and emotional regulation skills. Gao and Wang (2015) also confirmed through empirical analysis of older Chinese adults that physical exercise effectively improves subjective health perception and social functioning. For older adults, physical exercise serves both as a means of maintaining physical vitality and as a channel for building social connections and reaffirming self-worth. This synergy between the “physical” and the “social” dimensions makes exercise an important pathway for older adults to achieve social adaptation and sustain their social roles. Nevertheless, due to the lack of consensus on the duration, frequency, and intensity of physical exercise, further empirical evidence is needed to reinforce these conclusions.

### 4.2. The Mediating Role of Age Identity

The results of this study suggest that older adults’ age and identity play important mediating roles in the relationship between physical exercise and social adaptation. Hypotheses H2, H3, and H4 were validated, and these findings are highly consistent with existing studies and further enrich the theoretical mechanism by which physical exercise contributes to the social adaptation of older adults through the psycho-cognitive pathway. Guo and Huang (2025) reported that physical exercise significantly increased older adults’ age identity, i.e., the participants were more inclined to “think that they are not old yet”, and this rejuvenation of subjective perception led them to participate more actively and openly in social life (Diehl et al., 2014). From the perspective of the subjective life course, aging identity is the result of deep self-perception and social orientation, which represent how individuals define the status and meaning of self-aging in the aging stage. When older adults gain physical vitality and positive psychological feelings through exercise, they delay their identification with the role of “old age” and develop an attitude of “defying old age”, thus maintaining active social functioning and positive interpersonal interactions. This echoes the work of Levy and Myers (2004), who noted that older adults with younger subjective ages were more likely to engage in preventive health behaviors and showed better social adaptation in the long term.

In addition, the KHB mediation effect test in this study revealed that aging played a partial mediating role between physical exercise and social adaptation, accounting for approximately 11% of the total effect, indicating that, in addition to the direct physical mechanism, the change in psychological cognition also plays a bridging role in the improvement of social adaptation that cannot be ignored. This result suggests that, beyond the direct physiological mechanisms, psychological and cognitive changes also serve as an indispensable bridge in promoting social adaptation. This finding supports the view that social adaptation is essentially a proactive adjustment process through which individuals modify their behaviors, cognitions, and emotions in response to environmental changes (Rachmad, 2022). The value of physical exercise, therefore, lies not only in “moving the body,” but also in “feeling younger” and “connecting socially.” This aligns with the perspective proposed by Kim et al. (2016), who argued that physical exercise promotes active aging by strengthening self-esteem, enhancing psychological resilience, and fostering social identity, thereby facilitating self-renewal and reconnection among older adults. Similarly, Datan et al. (1987) emphasized that the stability of psychological health and physiological function is directly related to positive social adaptation. Taken together, the mediating mechanism of aging identity provides both theoretical and empirical support for understanding how physical exercise enhances social adaptation. It also highlights the practical implication that delaying the perception of aging should be considered a key objective in health promotion policies for older adults. Encouraging participation in physical exercise can stimulate older adults’ sense of agency and perceived youthfulness, fostering more active social engagement and contributing to the realization of genuine and successful aging.

### 4.3. Significant Heterogeneity in Adaptation Effects Among Different Groups of Older Adults

In further group experiments, physical exercise had a more pronounced effect on the enhancement of social adaptation for specific groups. Among them, the older adult groups with advanced age (≥80 years), low education level, low income, urban residence, no chronic disease, and old-age security showed greater social adaptation levels after participation in physical exercise, indicating obvious heterogeneous effects. Specifically, the regression coefficient of 0.757 was much greater in the 80 and older age groups than in the 60–69 age group (0.416), suggesting that as individuals grow older, they become more dependent on and more responsive to physical exercise when facing the challenges of deterioration of physical function and weakening of social ties and that exercise becomes an important mechanism for maintaining their social roles and psychological stability. Similarly, the adaptation effect of physical exercise was significantly greater in the less educated group than in the other groups (the regression coefficient was 0.892 in the illiterate group), which may be related to the inherent disadvantages of these groups in accessing information and social resources, with physical exercise serving as an important compensatory mechanism to offset deficiencies in cognition and social participation. Tang and Wang (2014) provided complementary evidence from another perspective, showing that older adults with higher educational attainment tend to possess richer social resources and stronger information-processing abilities, thereby exhibiting higher levels of social adaptation. Taken together, these findings suggest that educational level exerts a differentiated moderating effect on the relationship between physical exercise and social adaptation—highly educated older adults are more likely to adapt proactively by leveraging their social capital and cognitive advantages, whereas those with lower educational backgrounds achieve functional compensation for social integration through physical exercise.

At the income level, adaptation gains were also more pronounced in the lower-income group of older adults, demonstrating the sociological rule that “those with weaker resources are more sensitive to exercise interventions”. In contrast, the high-income group also had positive feedback, but the marginal effect was relatively weaker. The analysis of urban‒rural differences further confirms that older adults from urban residences have better access to exercise resources and socialization opportunities and that physical exercise is more effective in improving social adaptation, reflecting the empowering effect of fitness infrastructure and the social support environment. In addition, health status and old-age security play important roles in moderating the effects of exercise. Older adults without chronic diseases were the most affected by exercise (coefficient of 0.554), indicating that those who were healthy were better able to fully utilize the physical and mental benefits of exercise. In contrast, those who are ill are limited by their physical ability and health risks, and the benefits of exercise are impeded. In terms of old-age security, the effect of exercise is also more pronounced in the group with basic security, whereas the adaptive capacity of those who do not have security is even negative, suggesting that economic stress and life uncertainty may undermine the positive psychological and social functioning effects of exercise.

### 4.4. Practical Significance

This study focuses on the empirical analysis of the relationships among physical exercise, social adaptation of the elderly and age-related identity, which not only verifies the direct effect of physical exercise on enhancing the social adaptation of the elderly but also reveals the mediating mechanism of age-related identity and further highlights the differences in the effects among different groups. This series of findings has important theoretical and practical significance.

First, this study fills a gap in the empirical research on the relationship between physical exercise and the social adaptation ability of older adults and enriches the theoretical framework of physical exercise for social adaptation. Moreover, the introduction of the mediator variable “aging identity” more accurately disentangled the mechanism of the relationship between physical exercise and social adaptation in older adults and provided some basic ideas for the design of more related experiments. Second, the significant effects of physical exercise on older, less educated, lower-income, nonchronic-disease, and secure older adults, as analyzed by heterogeneity analysis, become important empirical evidence that can provide a direction for the future development of health intervention strategies for different populations at the level of community governance and public services. The evidence analyzed through differentiation has the theoretical value of providing precise empowerment for the accessibility and appropriateness of exercise resources. Finally, the finding that physical exercise not only enhances the physical fitness of older adults but also stimulates their sense of youthfulness, strengthens their social connections, and facilitates their integration into the community and social life is of great value to the social governance of older adults in the context of the increasing size of the aging population, as it improves their social functioning, psychological resilience, and subjective well-being. These findings are of great value to the important issues of social governance in the context of an expanding aging population and are of practical importance in promoting the in-depth integration of active aging and national fitness strategies.

### 4.5. Limitations and Future Research

Nevertheless, several limitations should be acknowledged. First, this study used a dichotomous variable to measure participation in physical exercise, which fails to capture more representative behavioral characteristics such as type, frequency, intensity, and duration of activity. Future studies could incorporate continuous or multidimensional indicators of exercise participation to improve the precision and real-world explanatory power of the analysis. Second, while this paper focuses on the mediating pathway of aging identity, physical exercise may also influence social adaptation through other psychosocial mechanisms, such as enhancing self-esteem, strengthening social support, or improving emotional well-being. Future research could adopt multiple-mediation models to examine these potential pathways in greater depth. Third, the study is based on cross-sectional data, which limits the ability to determine causal direction and temporal dynamics among the variables. Future research should employ longitudinal or panel data to establish the temporal order and strengthen causal inference. In addition, issues of model directness and sensitivity should be acknowledged. Despite controlling for key demographic and health-related factors, the associations identified in this study may still be subject to omitted-variable bias arising from unmeasured characteristics such as social support, depressive symptoms, community participation, personality traits, or neighborhood cohesion. These factors could jointly affect both physical exercise and social adaptation, thereby biasing the estimated coefficients. Furthermore, the relationship among the variables may be reciprocal rather than strictly unidirectional—for example, older adults with higher levels of social adaptation may be more likely to engage in physical exercise and develop a more positive aging identity. To address these concerns, future research should apply instrumental variable techniques, longitudinal structural equation modeling, or experimental designs to further validate the robustness and causal direction of the observed relationships.

## 5. Conclusions

This study examined the relationship between physical exercise and the social adaptation of older adults using data from the CLASS 2023 survey. The results confirmed that physical exercise significantly improves social adaptation, even after controlling for sex, age, education, income, and health status (β = 0.4523). As discussed above, physical exercise not only enhances physical fitness but also represents a composite behavior that integrates emotional regulation and social interaction, helping older adults maintain psychological stability and adapt to social changes. In addition, aging identity plays a significant partial mediating role in this relationship, accounting for approximately 11% of the total effect. This finding highlights the psychological value of physical exercise: by shaping a more positive aging identity, exercise indirectly enhances older adults’ social adaptability. The results also indicate heterogeneous effects across subgroups—those who are older (≥80 years) have lower education or income levels, live in urban areas, have no chronic disease, or enjoy old-age security benefits more from exercise, showing a greater improvement in adaptive capacity.

## Figures and Tables

**Table 1:** Variable selection and encoding (N = 8913).

Variable	Variable Type	Definition
Social Adaptation	Continuous Variable	Social Adjustment Scale Score
Physical Exercise	Categorical Variable	How often do you participate in physical exercise: less than once a month = 0, other = 1
aging identity	Continuous Variable	Difference between perceived and actual age at aging
Male	Categorical Variable	Female as reference category
Age	Continuous Variable	Difference between year of data collection and year of birth
Year of Education	Continuous Variable	Educational attainment converted to a yearly score
Nature of Account	Categorical Variable	Agricultural = 0, Urban = 1
Whether or not there is a spouse	Categorical Variable	Married with spouse = 1, Other = 0
Whether empty nester	Categorical Variable	Only 1 person permanently residing in the household = 1, Other = 0
Income Level	Continuous Variable	Logarithmic treatment of monthly income
Presence of Chronic Disease	Categorical Variable	No chronic disease = 0, any chronic disease = 1
Number of Children	Continuous Variable	Number of living children of respondents
With or without Social Security	Categorical Variable	Not enjoying any old-age benefits such as old-age pension, assistance pension, old-age allowance, old-age service subsidy, family planning family incentive grant, etc. = 0, at least one of them = 1

**Table 2:** Descriptive statistical results of the variables (N = 8913).

Variable	Full Sample (*n* = 8913)			Participate in Physical Exercise (*n* = 4312)			Nonparticipation in Physical Exercise (*n* = 4601)		
	Mean	Minimum	Maximum	Mean	Minimum	Maximum	Mean	Minimum	Maximum
Social Adaptation	24.32	8	40	24.63	8	40	23.98	8	40
Aging Identity	−0.06	−39	50	1.34	−34	47	−1.56	−39	50
Male	0.52	0	1	0.53	0	1	0.516	0	1
Age	71.29	59	98	70.66	59	95	71.97	59	98
Year of Education	6.55	0	16	6.96	0	16	6.11	0	16
Nature of Account	0.47	0	1	0.54	0	1	0.39	0	1
Whether or not there is a spouse	0.84	0	1	0.87	0	1	0.80	0	1
Whether empty nester	0.08	0	1	0.07	0	1	0.09	0	1
Income Level	9.52	4.61	12.90	9.58	4.60	11.98	9.46	4.61	12.90
Presence of Chronic Disease	0.83	0	1	0.85	0	1	0.82	0	1
Number of Children	2.18	0	9	2.05	0	9	2.32	0	8
With or without Social Security	0.93	0	1	0.94	0	1	0.91	0	1

**Table 3:** Correlation analysis among core variables.

Variable	Physical Exercise	Aging Identity	Social Adaptation
physical exercise	1		
Aging Identity	0.1179 ***	1	
Social Adaptation	0.0813 ***	0.1689 ***	1

Note: *** *p* < 0.01.

**Table 4:** OLS estimation results of the effects of physical exercise on the social adaptation of older adults.

Variable	Coefficient	Std. Error	t	*p* Value	95% CI Lower	95% CI Upper
physical exercise	0.452	0.086	5.27	*p* < 0.001	0.284	0.620
Age	−0.029	0.008	−3.39	0.001	−0.045	−0.012
Gender	−0.068	0.086	−0.79	0.432	−0.236	0.101
Log income	0.343	0.039	8.9	*p* < 0.001	0.267	0.418
Education	0.113	0.013	8.87	*p* < 0.001	0.088	0.139
Has Spouse	−0.147	0.149	−0.98	0.325	−0.439	0.146
Urban	0.173	0.099	1.75	0.08	−0.021	0.368
Empty nest	−0.396	0.194	−2.04	0.041	−0.777	−0.015
Social security	−0.116	0.164	−0.71	0.487	−0.408	0.439
Chronic disease	−0.116	0.116	−1	0.317	−0.339	0.107
Number of children	−0.014	0.044	−0.32	0.749	−0.102	0.073
Constant	22.232	0.747	29.77	*p* < 0.001	20.769	23.696

Note: N = 8913, R^2^ = 0.0447. Estimates are obtained using ordinary least squares (OLS) regression with robust standard errors. All subsequent regression models in Table 5, Table 6, Table 7, Table 8, Table 9 and Table 10 are estimated using the same specification.

**Table 5:** OLS estimation results of physical exercise on aging identity.

Variable	Coefficient	Std. Error	t	*p* Value	95% CI Lower	95% CI Upper
physical exercise	1.315	0.23	5.72	*p* < 0.001	0.865	1.766
Age	−0.733	0.023	−32.48	*p* < 0.001	−0.778	−0.689
Gender	0.186	0.231	0.42	0.420	−0.266	0.638
Log income	1.656	0.103	16.02	*p* < 0.001	1.453	1.856
Education	0.259	0.035	7.19	*p* < 0.001	0.183	0.319
Has Spouse	0.171	0.400	0.43	0.669	−0.613	0.956
Urban	−0.632	0.266	−2.38	0.017	−1.153	−0.111
Empty nest	−0.749	0.521	−1.44	0.151	−1.770	0.273
Social security	−0.395	0.211	−1.87	0.062	−1.263	0.473
Chronic disease	0.387	0.305	1.27	0.205	−0.211	−0.985
Number of children	−1.000	0.119	−8.38	*p* < 0.001	−1.234	−0.766
Constant	36.456	2.003	18.2	*p* < 0.001	32.529	40.384

Note: N = 8913, R^2^ = 0.0544.

**Table 6:** OLS estimation results of the effect of physical exercise on the social adaptation of older adults after the incorporation of aging identity.

Variable	Coefficient	Std. Error	t	*p* Value	95% CI Lower	95% CI Upper
physical exercise	0.403	0.086	4.71	*p* < 0.001	0.235	0.570
Aging Identity	0.038	0.004	9.57	*p* < 0.001	0.030	0.045
Age	−0.001	0.009	−0.1	0.919	−0.018	0.016
Gender	−0.074	0.086	−0.87	0.384	−0.242	0.093
Log income	0.281	0.039	7.22	*p* < 0.001	0.204	0.357
Education	0.104	0.013	8.04	*p* < 0.001	0.079	0.129
Has Spouse	−0.153	0.148	−1.03	0.302	−0.444	0.138
Urban	0.198	0.099	2.00	0.045	0.004	0.391
Empty nest	−0.368	0.193	−1.90	0.057	−0.747	0.011
Social security	0.131	0.164	0.79	0.427	−0.191	0.452
Chronic disease	−0.130	0.113	−1.15	0.250	−0.352	0.092
Number of children	0.023	0.044	0.53	0.599	−0.064	0.111
Constant	20.861	0.757	27.57	*p* < 0.001	19.377	22.344

Note: N = 8913, R^2^ = 0.2565.

**Table 7:** KHB results of the mediation effect decomposition.

Model Type	Coefficient	Std. Error	Z	*p*	95% CI Lower	95% CI Upper
Reduced	0.452	0.085	5.30	<0.001	0.286	0.620
Full	0.403	0.085	4.71	<0.001	0.236	0.570
Diff	0.050	0.011	4.91	<0.001	0.029	0.069

**Table 8:** OLS estimation results of social adaptation in older adults via the sample replacement method.

Variable	Coefficient	Std. Error	t	*p* Value	95% CI Lower	95% CI Upper
Physical Exercise	0.554	0.096	5.76	<0.001	0.365	0.742
Aging Identity	0.060	0.004	14.85	<0.001	0.052	0.068
Age	−0.010	0.009	−1.07	0.285	−0.027	0.008
Gender	0.043	0.093	0.46	0.646	−0.140	0.226
Education	0.041	0.013	3.2	0.001	0.016	0.068
Has Spouse	−0.123	0.132	−0.93	0.352	−0.380	0.135
Urban	0.321	0.093	3.44	0.002	0.120	0.525
Empty nest	−0.142	0.181	−0.78	0.435	−0.498	0.214
Social security	−0.089	0.173	−0.51	0.609	−0.429	0.252
Chronic disease	−0.617	0.117	−5.26	<0.001	−0.847	−0.387
Number of children	0.099	0.043	2.29	0.022	0.017	0.182
Constant	24.643	0.659	37.39	<0.001	23.351	25.935

Note: N = 8487, R^2^ = 0.0489.

**Table 9:** KHB decomposition results of the sample replacement method.

Model Type	Coefficient	Std. Error	Z	*p*	95% CI Lower	95% CI Upper
Reduced	0.6524	0.0959	6.81	0.000	0.4646	0.8403
Full	0.5538	0.0961	5.76	0.000	0.3654	0.7421
Difference	0.0988	0.0168	5.86	0.000	0.0657	0.1317

**Table 10:** Ordered logit estimation results of the effect of physical exercise on the social adaptation of older adults.

Variable	OR	95% CI	*p* Value
Physical Exercise	1.224	1.137–1.319	<0.001
Aging Identity	1.013	1.009–1.016	<0.001
Age	0.999	0.992–1.007	0.879
Gender	0.984	0.913–1.060	0.669
Income	1.154	1.116–1.193	<0.001
Education	1.049	1.037–1.061	<0.001
Has Spouse	0.942	0.830–1.071	0.362
Urban	1.070	0.990–1.156	0.084
Empty nest	0.818	0.693–0.965	0.018
Social security	0.902	0.838–1.127	0.705
Chronic disease	0.986	0.891–1.091	0.781
Number of children	1.000	0.962–1.038	0.980

Note: N = 8913, LRχ^2^ = 451.21, pseudo R^2^ = 0.0092.

**Table 11:** Bootstrap analysis of the mediating variables after the incorporation of aging identity.

Effect Type	Estimate	Boot SE	z	*p* Value	95% CI Lower	95% CI Upper
Aging Identity	0.0499	0.0103	4.81	<0.001	0.0292	0.0697

**Table 12:** Differences among older adult groups of different ages and educational levels.

Variable	Age (Year)			Educational Level		
	60~69	70~79	≥80	Illiterate	Primary and Secondary School	Tertiary and Above
Physical Exercise	0.416 *** (0.130)	0.479 *** (0.127)	0.757 *** (0.244)	0.892 *** (0.183)	0.323 *** (0.185)	0.531 ** (0.242)
Control Variables	Yes	Yes	Yes	Yes	Yes	Yes
constant term (math.)	26.45 *** (1.854)	19.27 *** (1.932)	8.878 *** (3.546)	20.43 *** (1.491)	22.95 *** (0.893)	28.45 *** (2.274)
Number of observation samples	3586	4376	932	1544	6211	1158
R^2^	0.058	0.026	0.077	0.033	0.021	0.865

Note: Standard deviation in parentheses, *** *p* < 0.01, ** *p* < 0.05, The same notation applies to the following tables.

**Table 13:** Differences among older adult groups with different income levels and urban‒rural distributions.

Variable	Income Level		Registered Residence	
	High-Income Groups	Low-Income Groups	Urban Residence	Nonurban Residence
Physical Exercise	0.241 *** (0.118)	0.663 *** (0.124)	0.536 *** (0.125)	0.399 *** (0.117)
Control Variables	Yes	Yes	Yes	Yes
constant term (math.)	27.53 *** (0.906)	24.20 *** (0.903)	23.20 *** (1.126)	21.42 *** (1.031)
Number of observation samples	4494	4419	4163	4750
R^2^	0.036	0.032	0.073	0.013

Note: Income levels are divided by the median income of the sample as a boundary, taking the two ends of the spectrum to make judgments. Standard deviations are in parentheses. *** *p* < 0.01, ** *p* < 0.05. The same notation applies to the following tables.

**Table 14:** Differences among older adult groups with different health conditions and pension security statuses.

Variable	Chronic Disease		Old-Age Security	
	No	Yes	No	Yes
Physical Exercise	0.554 *** (0.205)	0.429 *** (0.0945)	−0.829 ** (0.382)	0.562 *** (0.0876)
Control Variables	Yes	Yes	Yes	Yes
constant term (math.)	20.36 *** (1.775)	22.40 *** (0.828)	31.35 *** (3.950)	21.81 *** (0.754)
Number of observation samples	1493	7420	652	8261
R^2^	0.066	0.045	0.053	0.049

## Data Availability

Our study used data from the Chinese Longitudinal Aging Social Survey (CLASS), administered by the National Survey Research Center at Renmin University of China. These data are not publicly available and were obtained under license for the current study. Access to CLASS data can be requested from the National Survey Research Center at Renmin University of China (http://class.ruc.edu.cn) with permission.

## References

[B1-behavsci-15-01491] Baron R. M., Kenny D. A. (1986). The moderator–mediator variable distinction in social psychological research: Conceptual, strategic, and statistical considerations. Journal of Personality and Social Psychology.

[B2-behavsci-15-01491] Barrett A. E., Barbee H. (2022). The subjective life course framework: Integrating life course sociology with gerontological perspectives on subjective aging. Advances in Life Course Research.

[B3-behavsci-15-01491] Caspersen C. J., Powell K. E., Christenson G. M. (1985). Physical activity, exercise, and physical fitness: Definitions and distinctions for health-related research. Public Health Reports.

[B4-behavsci-15-01491] Chen B. (2008). Social adaptation of the urban elderly in an aging era. Journal of Social Sciences.

[B5-behavsci-15-01491] Chen S.-P., Li S. Z., Yan Z. L. (2006). Research on mechanism of exercise persistence based on sport commitment theory. China Sport Science.

[B6-behavsci-15-01491] Datan N., Rodeheaver D., Hughes F. (1987). Adult development and aging. Annual Review of Psychology.

[B7-behavsci-15-01491] de Souto Barreto P. (2009). Exercise and health in frail elderly people: A review of randomized controlled trials. European Review of Aging and Physical Activity.

[B8-behavsci-15-01491] Diehl M., Wahl H. W., Barrett A. E., Brothers A. F., Miche M., Montepare J. M., Westerhof G. J., Wurm S. (2014). Awareness of aging: Theoretical considerations on an emerging concept. Developmental Review.

[B9-behavsci-15-01491] Folstein M. F., Folstein S. E., McHugh P. R. (1975). Mini-mental state: A practical method for grading the cognitive state of patients for the clinician. Journal of Psychiatric Research.

[B10-behavsci-15-01491] Gai W., Mei J. (2024). Influence of sports training on athletes’ social adaptability. Shandong Sports Science.

[B11-behavsci-15-01491] Gao L., Wang L. (2015). Investigation of the relationship between physical exercise and self-reported health among the elderly. Journal of Wuhan Institute of Physical Education.

[B12-behavsci-15-01491] Gordon B. R., McDowell C. P., Lyons M., Herring M. P. (2020). Resistance exercise training for anxiety and worry symptoms among young adults: A randomized controlled trial. Scientific Reports.

[B13-behavsci-15-01491] Grad F. P. (2002). The preamble of the Constitution of the World Health Organization. Bulletin of the World Health Organization.

[B14-behavsci-15-01491] Grech H. (2019). Impact of forced migration on communication and social adaptation. Folia Phoniatrica et Logopaedica.

[B15-behavsci-15-01491] Guo K., Huang Q. (2025). Where does “not accepting old age” come from? The role path of physical exercise in aging identity among Chinese older adults. Sports Studies Research.

[B16-behavsci-15-01491] He J., Zhao Y., Zhang H., Lin Z. (2021). Orthorexia nervosa is associated with positive body image and life satisfaction in Chinese older adults: Evidence for a positive psychology perspective. International Journal of Eating Disorders.

[B17-behavsci-15-01491] Hu B., Wang Z. (2017). A review of research on physical exercise and mental health. Chinese School Physical Education (Higher Education).

[B18-behavsci-15-01491] Jiang C., Shi J. (2024). The impact of aging attitudes on the mental health of older adults: An empirical analysis based on the perspective of three-dimensional digital integration. Beijing Social Sciences.

[B19-behavsci-15-01491] Kim J., Chun S., Heo J., Lee S., Han A. (2016). Contribution of leisure-time physical activity on psychological benefits among elderly immigrants. Applied Research in Quality of Life.

[B20-behavsci-15-01491] Levy B. R., Myers L. M. (2004). Preventive health behaviors influenced by self-perceptions of aging. Preventive Medicine.

[B21-behavsci-15-01491] Li C., Ning G., Xia Y. (2023). Does exercise participation promote happiness? Mediations and heterogeneities. Frontiers in Public Health.

[B22-behavsci-15-01491] Li X., Li C. (2025). Promoting healthy aging: Physical activity and its dual effects on physical health and cognitive function in Chinese older adults. Frontiers in Public Health.

[B23-behavsci-15-01491] Liddell T. M., Kruschke J. K. (2018). Analyzing ordinal data with metric models: What could possibly go wrong?. Journal of Experimental Social Psychology.

[B24-behavsci-15-01491] Liu W., Qi Z. (2024). Localization interpretation of healthy aging and the transformation path of national fitness technology services to “adapt to aging”. Journal of Xi’an Sport University.

[B25-behavsci-15-01491] Liu X., Cao X., Gao W. (2022). Does low self-esteem predict anxiety among Chinese college students?. Psychology Research and Behavior Management.

[B26-behavsci-15-01491] Ma W. (2017). Research on the Relationship between Sports Participation and Personality Characteristics and Social Adaptability of College Students. Journal of Harbin Institute of Physical Education.

[B27-behavsci-15-01491] Ma X., Li X., Che L., Dong J. (2025). Influence of physical exercise on activities of daily living in older adults: An empirical analysis based on propensity score matching and difference-in-differences. Frontiers in Public Health.

[B28-behavsci-15-01491] McAuley E., Elavsky S., Motl R. W., Konopack J. F., Hu L., Marquez D. X. (2005). Physical activity, self-efficacy, and self-esteem: Longitudinal relationships in older adults. The Journals of Gerontology Series B: Psychological Sciences and Social Sciences.

[B29-behavsci-15-01491] National Bureau of Statistics of China (2021). Bulletin of the seventh national population census (No. 5)-Population age composition. China Statistics.

[B30-behavsci-15-01491] Netz Y., Wu M. J., Becker B. J., Tenenbaum G. (2005). Physical activity and psychological well-being in advanced age: A meta-analysis of intervention studies. Psychology and Aging.

[B31-behavsci-15-01491] Paterson D. H., Warburton D. E. R. (2010). Physical activity and functional limitations in older adults: A systematic review related to Canada’s physical activity guidelines. International Journal of Behavioral Nutrition and Physical Activity.

[B32-behavsci-15-01491] Putnam R. D. (1995). Bowling alone: America’s declining social capital. Journal of Democracy.

[B33-behavsci-15-01491] Rachmad Y. E. (2022). Social adaptation theory.

[B34-behavsci-15-01491] Robbert R. (1983). Adaptation to aging: The maintenance of self.

[B35-behavsci-15-01491] Spencer H. (1898). Principles of sociology.

[B36-behavsci-15-01491] Tang D., Wang D. (2014). Anxiety of elderly adults: Level and influencing factors. Studies of Psychology and Behavior.

[B37-behavsci-15-01491] Tsai M. J. (2017). Revisiting communicative competence in augmentative and alternative communication. Folia Phoniatrica et Logopaedica.

[B38-behavsci-15-01491] VanKim N. A., Nelson T. F. (2013). Vigorous physical activity, mental health, perceived stress, and socializing among college students. American Journal of Health Promotion.

[B39-behavsci-15-01491] Zhou Z., Guo K., Guo S., Chen L. (2024). Relationship between physical exercise and college students’ social adaptation: The chain mediating role of self-esteem and peer attachment. Frontiers in Psychology.

